# Supplementary material for: A haemagglutination test for rapid detection of antibodies to SARS-CoV-2
Source: Nat Commun. 2021 Mar 29;12:1951. doi: 10.1038/s41467-021-22045-y (PMC8007702; doi:10.1038/s41467-021-22045-y)
Supplement: Supplementary file 1 — Supplementary Information [file 41467_2021_22045_MOESM1_ESM.pdf]

SUPPLEMENTARY INFORMATION

Supplementary note: DNA sequence of codon optimised cDNA encoding IH4-RBD

ATGGGATGGTCATGTATCATCCTTTTTCTAGTAGCAACTGCAACCGGTGTTCATAGCCAGGTC
CAGCTGCAAGAGTCTGGCGGAGGATCTGTTCAGGCTGGCGGAAGCCTGAGACTGAGCTGTGT
GGCCAGCGGCTACACCGATAGCACATACTGCGTCGGCTGGTTCAGACAGGCCCCTGGCAAAG
AGAGAGAGGGCGTCGCCAGAATCAACACCATCAGCGGCAGACCTTGGTACGCCGACTCTGTG
AAGGGCAGATTCACAATCAGCCAGGACAACAGCAAGAACACCGTGTTTCTGCAGATGAACA
GCCTGAAGCCAGAGGACACCGCCATCTACTACTGCACCCTGACCACCGCCAACAGCAGAGGC
TTTTGTTCCGGCGGCTACAACTACAAAGGCCAGGGCACCCAAGTGACCGTGTCTAGCGCGTC
GACCGGCTCTGGCGGCAGCGGCAACATCACCAATCTGTGCCCTTTCGGCGAGGTGTTCAACG
CCACCAGATTTGCCAGCGTGTACGCCTGGAACCGGAAGAGAATCAGCAACTGCGTGGCCGAC
TACAGCGTGCTGTACAATAGCGCCAGCTTCAGCACCTTCAAGTGCTACGGCGTGTCCCCTACC
AAGCTGAACGACCTGTGCTTCACCAATGTGTACGCCGACAGCTTCGTGATCAGAGGCGACGA
AGTTCGGCAGATCGCTCCTGGACAGACAGGCAAGATCGCCGATTACAACTACAAGCTGCCCG
ACGACTTCACCGGCTGCGTGATCGCCTGGAATAGCAACAACCTGGACAGCAAAGTCGGCGGC
AACTACAACTACCTGTACCGGCTGTTCCGGAAGTCCAACCTGAAGCCTTTCGAGCGGGACAT
CAGCACCGAGATCTATCAGGCCGGCAGCACCCCTTGTAATGGCGTGGAAGGCTTCAACTGCT
ACTTCCCACTGCAGTCCTACGGCTTTCAGCCTACAAACGGCGTGGGCTACCAGCCTTATAGAG
TGGTGGTGCTGAGCTTCGAACTGCTGCATGCCCCTGCTACCGTGTGCGGCCCTAAAAACACC
ATCACCACCACCATTGA

Supplementary Table 1. Fifteen divalent RBD binding molecules, composed of 11 human monoclonal antibodies, 2 Fc linked nanobodies, an anti-His-tag murine monoclonal antibody, and ACE2-Fc were titrated in the HA Test. Three additional monoclonal antibodies to other regions of the Spike protein, EW-8B, EW-9C and FJ-1C, provided negative controls. One divalent nanobody VHH72-Fc and one monoclonal antibody FD-5D specific for the RBD failed to agglutinate red cells with the IH4-RBD reagent. Antibodies were made up at 20 μg/mL (1000 ng in 50 μL) and doubling dilutions performed with 50 μL, 50 μL of O- whole blood (1:40) was added, followed by 50 μL of IH4-RBD (2 μg/mL containing 100 ng). The results are expressed as the amount of antibody added per well (rounded to a whole number) at the HAT endpoint of loss of teardrop. These results have been reproduced at least twice.

Mab or divalent Nanobody	HAT endpoint titre (ng/well)	Reference
CR3022	2	(Huo, Zhao, et al., 2020; ter Meulen et al., 2006)
EY-6A	16	(Zhou et al., 2020)
VHH-72-Fc	Negative	(Wrapp et al., 2020)
FI-4A	16	(Huang et al., 2020)
ACE2-Fc	4	(Huo, Le Bas, et al., 2020)
FI-3A	8	(Huang et al., 2020)
C121	8	(Robbiani et al., 2020)
H11-H4-Fc	16	(Huo, Le Bas, et al., 2020)
FD-5D	Negative	(Huang et al., 2020)
FD-11A	31	(Huang et al., 2020)
FN-12A	31	(Huang et al., 2020)
FJ-10B	62	(Huang et al., 2020)
FM-7B	62	(Huang et al., 2020)
S309	125	(Pinto et al., 2020)
Anti-His-tag	62.5	Invitrogen MA1-21315
EW-8B non-RBD anti Spike	Neg Control	(Huang et al., 2020)
EW-9C non-RBD anti Spike	Neg Control	(Huang et al., 2020)
FJ-1C anti NTD	Neg Control	(Huang et al., 2020)

Supplementary Table 2. Fifty-two samples from 24 donors who were sampled repeatedly during the first five days in hospital; assay repeated 34 days after the measurements in Table 1. Sixteen of 24 showed a rise in titre.

		Day				
**No**	**Case**	**1**	**2**	**3**	**4**	**5**
**1**	**C2**			**40**		**160**
**2**	**C6**			**0**		**80**
3	C7	0				0
**4**	**C9**	**0**				**1280**
5	C10	**160**		**320**		
**6**	**C23**	**0**		**80**		**320**
**7**	**S5**	40		40		
**8**	**S6**	**1280**		**2560**		**2560**
**9**	**S7**	**80**				**1280**
**10**	**S12**	**320**		**640**		
**11**	**S13**	**0**		**0**		**160**
12	S14			**640**		**1280**
13	S17			1280		1280
**14**	**S20**	**80**		**320**		
**15**	**S31**	**0**				**640**
**16**	**S41**	**640**		**1280**		
**17**	**S43**			**40**		**80**
**18**	**S48**			1280		1280
**19**	**M9**			**40**		**160**
20	M11	0		0		0
21	M13			0		0
22	M14			163840		163840
**23**	**M25**	**40**				**320**
24	M30			160		160

Supplementary Table 3. Monoclonal Antibodies FI-3A Class 1 (Huang et al., 2020), C121 Class 2 (Robbiani et al., 2020), FD-11A Class 3 (Huang et al., 2020) and CR3022 class 4 (Huo, Zhao, et al., 2020) compared on three sources of O-ve Red cells (A-C) performed in quadruplicates (four samples run in parallel in the same experiment) (1-4), read by two independent scorers. The "Class" of the antibody refers to the four characterized binding sites on the RBD (Barnes et al., 2020). The values marked in red show the disagreements between scorers, that never differed by more than one doubling dilution (5/108 measurements). The values in the table refer to the titration end point (loss of teardrop) as number of doubling dilutions of the plasma sample starting with 1=1:40.

	FI-3A Class 1			C121 Class 2			FD-11A Class 3			CR3022 Class 4			040 -ve Control anti Ebola		
O-ve Donor:	**A**	**B**	**C**	**A**	**B**	**C**	**A**	**B**	**C**	**A**	**B**	**C**	**A**	**B**	**C**
1	8	9	8	7	8	7	4	5	4	8	9	8	0	0	0
2	8	10	8	7	8	7	4	5	4	8	9	8	0	0	0
3	8	9	8	7	8	7	4	4	4	8	9	8	0	0	0
4	8	9	8	7	8	7	4	4	4	8	9	8	0	0	0

	FI-3A Class 1			C121 Class 2			FD-11A Class 3			CR3022 Class 4			040 -ve Control anti Ebola		
O-ve Donor:	**A**	**B**	**C**	**A**	**B**	**C**	**A**	**B**	**C**	**A**	**B**	**C**	**A**	**B**	**C**
1	8	9	8	7	8	7	4	5	4	8	9	8	0	0	0
2	8	9	8	7	8	7	4	5	4	8	9	8	0	0	0
3	8	9	8	7	8	7	4	5	4	8	9	8	0	0	0
4	8	9	8	7	8	7	4	4	4	8	9	8	0	0	0

Supplementary Table 4. Four plasma samples were selected for a range of titres based on measurements done the week before: high titre (9DD = 1:10,240 Serum 9A), Medium Titre (6DD = 1: 1,280, Serum 11B), Low Titre (3DD – 1:160, Serum 11G) and a negative serum (10G) were titrated on 3 sources of O-ve Red cells (A-C) performed in quadruplicates (four samples run in parallel in the same experiment) (1-4), and read by two independent scorers. The values in the table refer to the titration end point (loss of teardrop) as number of doubling dilutions of the plasma sample starting with 1=1:40.

	Serum 9A			Serum 11B			Serum 11G			Serum 10G -ve Control		
O-ve Donor:	**A**	**B**	**C**	**A**	**B**	**C**	**A**	**B**	**C**	**A**	**B**	**C**
1	9	9	9	5	7	6	3	4	3	0	0	0
2	9	9	9	5	7	6	3	4	3	0	0	0
3	9	9	9	6	7	6	3	4	3	0	0	0
4	9	9	9	5	7	6	3	4	3	0	0	0

	Serum 9A			Serum 11B			Serum 11G			Serum 10G -ve Control		
O-ve Donor:	**A**	**B**	**C**	**A**	**B**	**C**	**A**	**B**	**C**	**A**	**B**	**C**
1	9	9	9	5	7	6	3	4	3	0	0	0
2	9	9	9	5	7	6	3	4	3	0	0	0
3	9	9	9	5	6	6	3	4	3	0	0	0
4	9	9	9	5	6	6	3	4	3	0	0	0

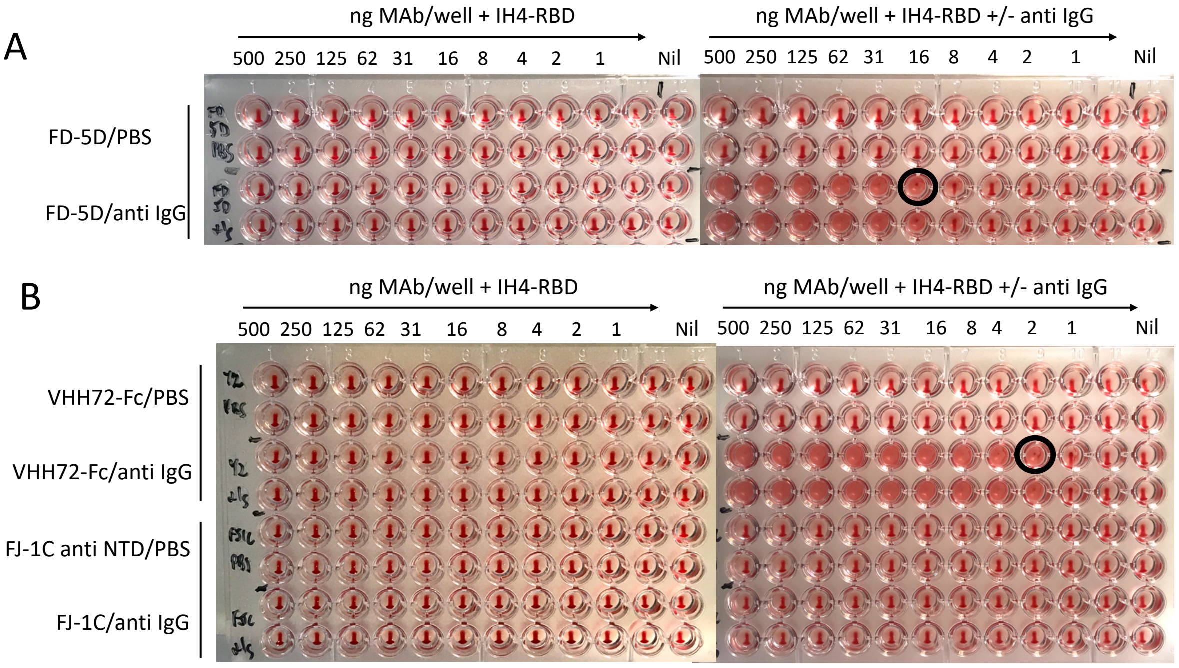

Supplementary Figure 1. A, B) Two MAbs or Nanobody-Fc reagents of 13 tested specific for RBD failed to cross-link red cells but could be developed with an anti IgG reagent. Antibodies were titrated from 500 ng/well to 1 ng/well in 50 µL, 1:40 red cells were added in 50 µL, followed by 100 ng/well IH4-RBD in 50 µL, and incubated for 1 hour. The labelled red cells were then resuspended, and either 50 µL PBS (L hand plates), or Sigma Clone GG5 anti human IgG 1:100 in 50 µL (R hand plates). Red cells were allowed to settle for one hour, the plate tilted for 30s and photographed. The experiment was repeated with the same result.

A

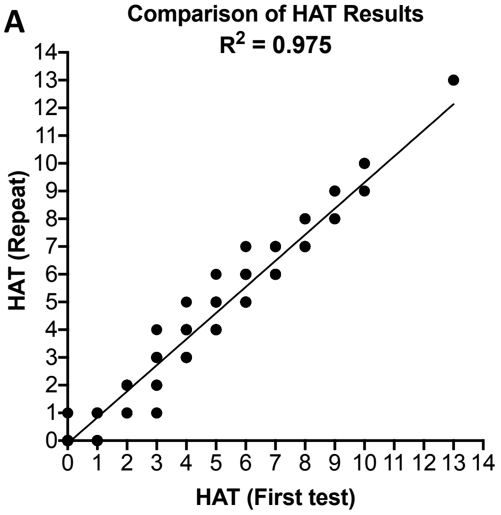

Slope = 0.94 (95% CI 0.92 - 0.96)

Suggests second set has tendency
to detect ~1 doubling dilution (DD) lower titres

230/232 (99%) were within 1 DD

B Titration in HAT by doubling dilution (Repeat)

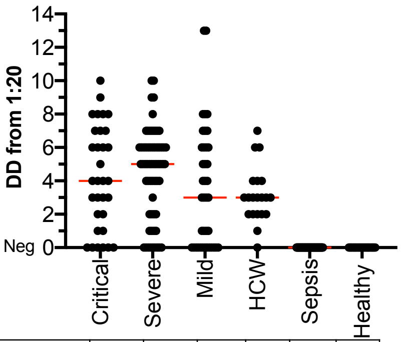

	Critical	Severe	Mild	HCW	Sepsis	Healthy
N samples	32	62	39	20	54	25
N donors	24	48	32		43	

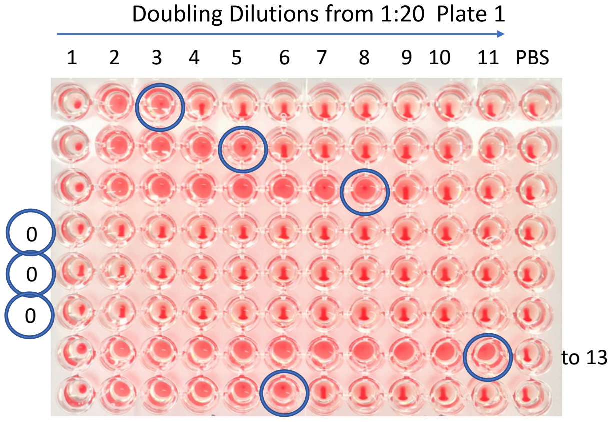

Donors were grouped according to clinical criteria; HCW = Front Line Health Care Workers; Sepsis = Samples with patients with Sepsis prior to Covid-19 pandemic; Healthy = Healthy volunteers prior to Covid-19; DD 1 = 1:40. Actual Titre = 20 x 2^DD.

C

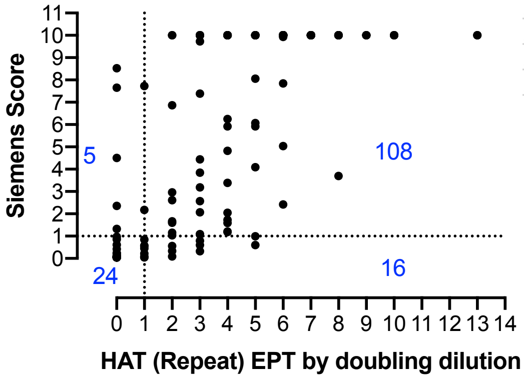

	HAT +ve	HAT -ve	Total	Fraction
Siemens +ve	108	5	113	0.96
Siemens -ve	16	24	40	0.40
Total	124	29	153	0.81
Fraction	0.87	0.83	0.74	

P value and statistical significance	
Test	Fisher's exact test
P value	<0.0001
P value summary	****
One- or two-sided	Two-sided

Sensitivity (Sn) HAT 81%, Sn Siemens 74%
HAT still more sensitive than Siemens

Specificity 100% for HAT in these samples

Supplementary Figure 2. A) The 232 samples from figure 5 in the main paper were titrated a second time 34 days after the first measurement, as described for figure 4 (Methods). The values for the end point doubling dilution were compared and a correlation coefficient, and slope calculated (Prism v8).

B) The collection included 32 samples from 24 Critical patients, 62 samples from 48 Severe, 39 samples from 32 Mild, 20 single samples from health care workers (HCW), 54 samples from 43 patients with unrelated sepsis in the pre-Covid-19 era, 25 samples from healthy unexposed controls. Median is shown.

C) Comparison to Siemens result (anti RBD) with HAT Titre by doubling dilution for 153 samples from Critical, Severe, Mild and HCW PCR+ve donors. The sensitivity of the HAT in this repeat was 81%, v 86% in the first test.

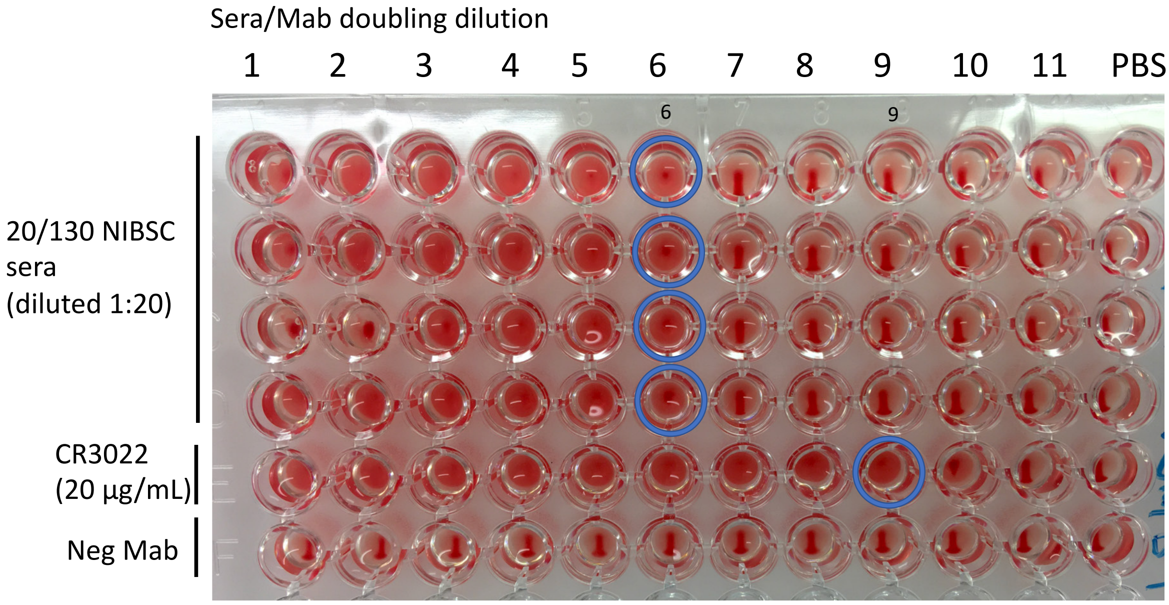

Supplementary Figure 3. Titration of NIBSC sera 20/130 in hemagglutination test. The Serum 20/130 was diluted 1:20 in PBS, CR3022 and Neg Control Mab (040 to Ebola GP) were diluted to 20ug/ml in PBS. 50ul aliquots were placed in column 1 and doubling dilutions made from columns 1 – 11. 20/130 (1:20) had endpoint at 6 Doubling Dilutions (1:1280), CR3022 (20ug/ml) reached its endpoint at 9 Doubling Dilutions (= 2 ng/well). The experiment was repeated with the same result.

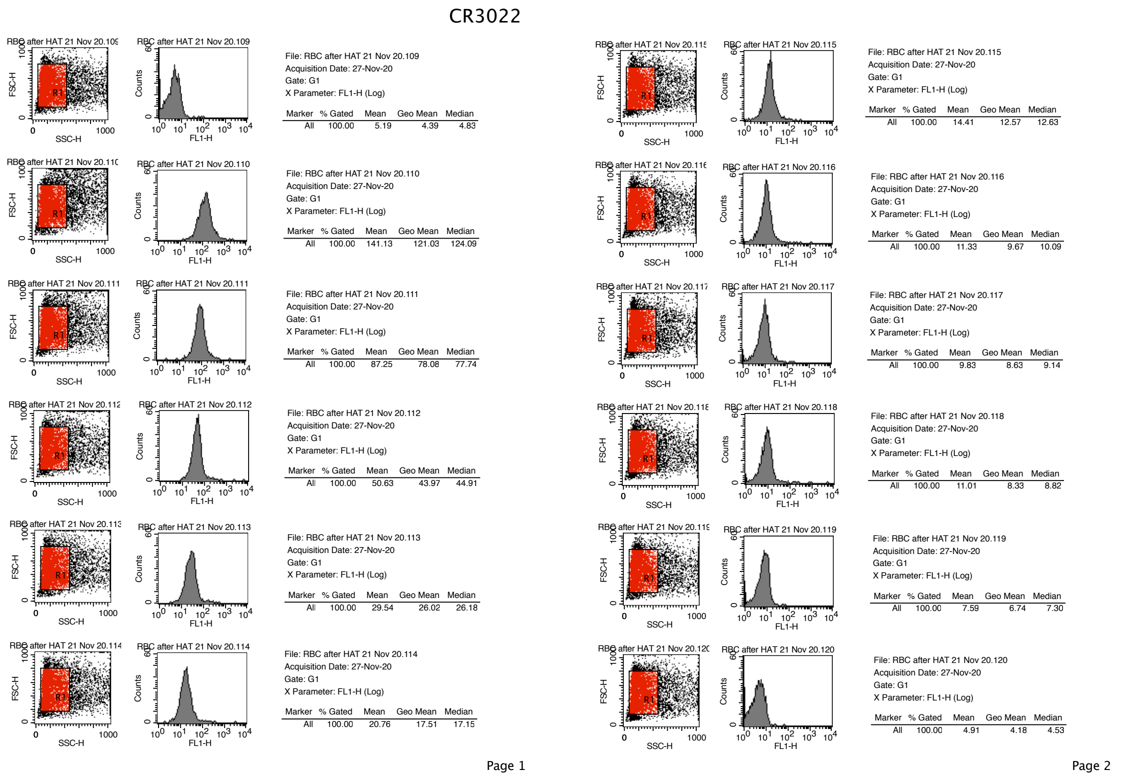

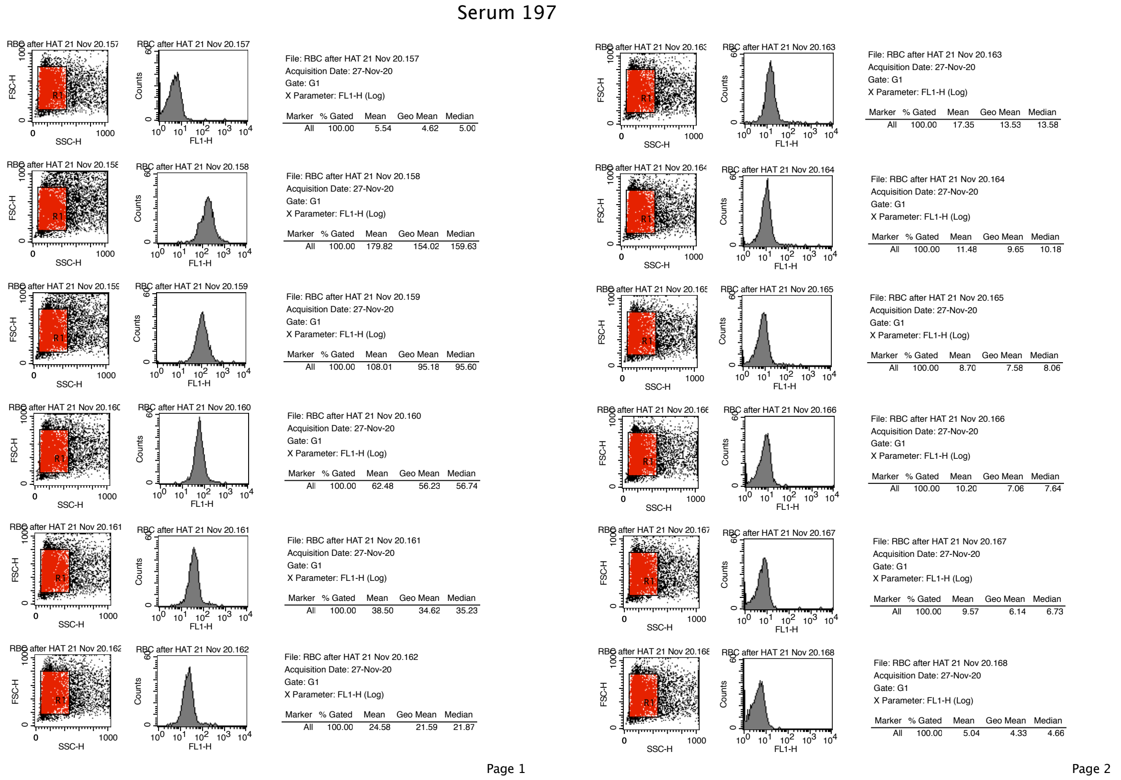

Supplementary Figure 4. Flow cytometry data used for Figure 3C, showing the gating strategy, the histograms with the actual scales (Fluorescence intensity vs cell numbers), and the statistics for each of the histograms used.

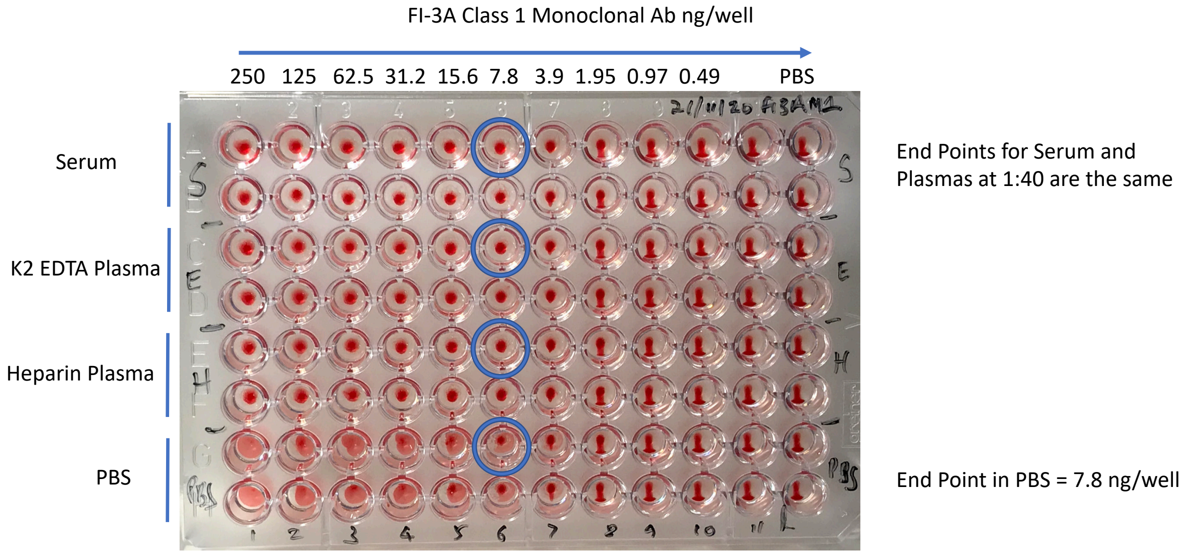

Supplementary Figure 5. The presence of serum or plasma does not alter the titration end-points in the HAT assay. Monoclonal Antibody FI-3A (Class 1) was diluted to 20 µg/ml in 1:20 serum or K2EDTA or Heparin Plasma. Doubling Dilutions in 1: 20 serum/plasma were prepared, and 25 µL of 1:20 whole blood in PBS added to all wells. 50 µL of IH4-RBD (2 µg/mL) was added to all wells. After 1 hr incubation the plate was tilted for ~30 s and photographed. This set up replicates the arrangement in the screening assay in Figure 4.

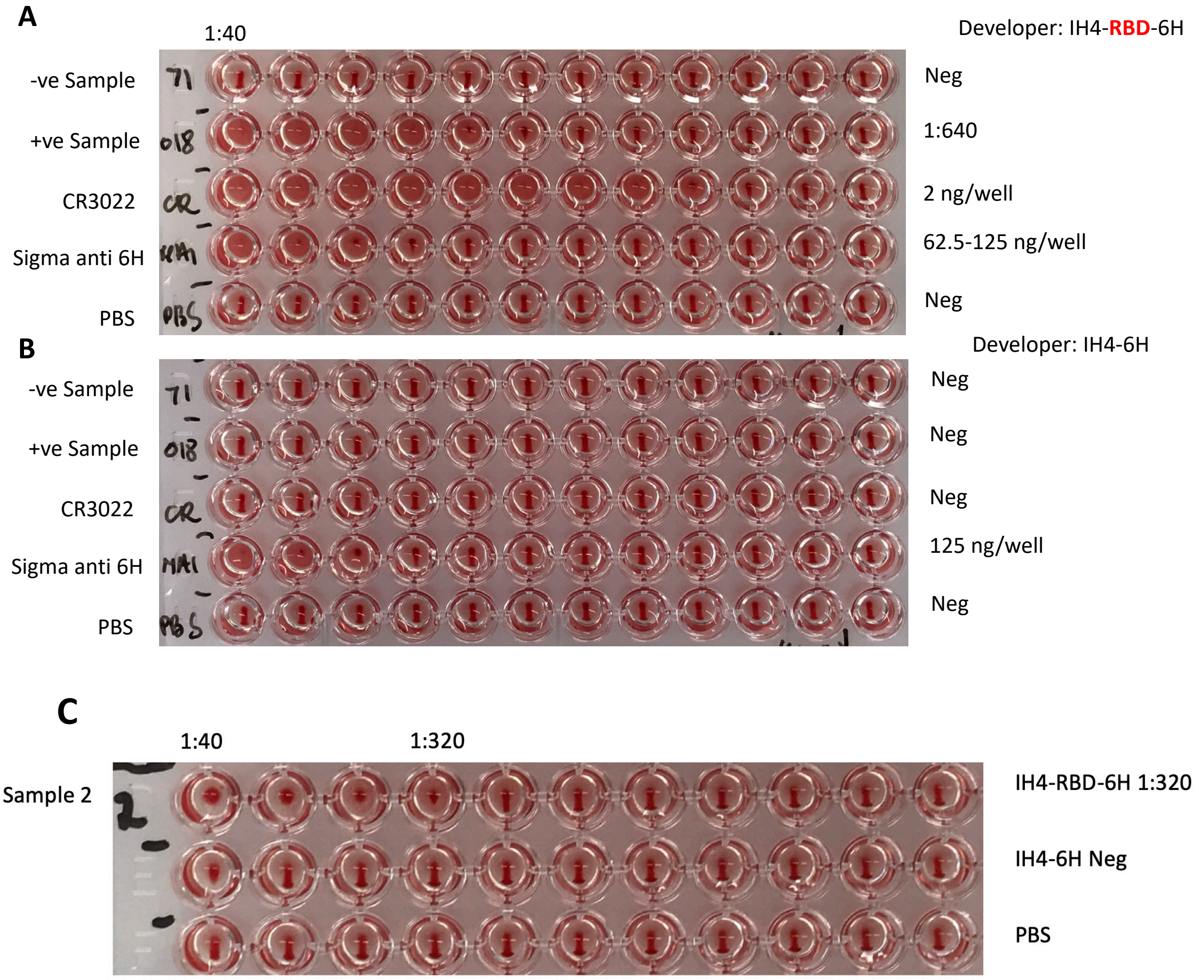

Supplementary Figure 6. A, B) a known negative plasma (71) and the strongest positive plasma sample from figure 3 (018 RBD EPT 11,286) were titrated in the HAT assay in comparison to monoclonal antibody CR3022, the anti 6H monoclonal antibody MA1-21315 (Invitrogen) or PBS as a negative control. The wells were developed with 100 ng/well of IH4-RBD-6H or IH4-6H. C) With these controls in place sample 002 (RBD EPT <50) from Figure 3 was titrated and developed with either IH4-RBD-6H or IH4-6H.
