# Supplementary material for: A haemagglutination test for rapid detection of antibodies to SARS-CoV-2
Source: Nat Commun. 2021 Mar 29;12:1951. doi: 10.1038/s41467-021-22045-y (PMC8007702; doi:10.1038/s41467-021-22045-y)
Supplement: Supplementary file 2 — Reporting Summary [file 41467_2021_22045_MOESM2_ESM.pdf]

nature research

Corresponding author(s): Etienne Joly

Last updated by author(s): 17 december 2020

Reporting Summary

Nature Research wishes to improve the reproducibility of the work that we publish. This form provides structure for consistency and transparency in reporting. For further information on Nature Research policies, see our Editorial Policies and the Editorial Policy Checklist.

Statistics

For all statistical analyses, confirm that the following items are present in the figure legend, table legend, main text, or Methods section.

n/a	Confirmed	
☐	☒	The exact sample size (*n*) for each experimental group/condition, given as a discrete number and unit of measurement
☐	☒	A statement on whether measurements were taken from distinct samples or whether the same sample was measured repeatedly
☐	☒	The statistical test(s) used AND whether they are one- or two-sided<br>*Only common tests should be described solely by name; describe more complex techniques in the Methods section.*
☒	☐	A description of all covariates tested
☒	☐	A description of any assumptions or corrections, such as tests of normality and adjustment for multiple comparisons
☐	☒	A full description of the statistical parameters including central tendency (e.g. means) or other basic estimates (e.g. regression coefficient) AND variation (e.g. standard deviation) or associated estimates of uncertainty (e.g. confidence intervals)
☐	☒	For null hypothesis testing, the test statistic (e.g. *F*, *t*, *r*) with confidence intervals, effect sizes, degrees of freedom and *P* value noted<br>*Give P values as exact values whenever suitable.*
☒	☐	For Bayesian analysis, information on the choice of priors and Markov chain Monte Carlo settings
☒	☐	For hierarchical and complex designs, identification of the appropriate level for tests and full reporting of outcomes
☒	☐	Estimates of effect sizes (e.g. Cohen's *d*, Pearson's *r*), indicating how they were calculated

Our web collection on statistics for biologists contains articles on many of the points above.

Software and code

Policy information about availability of computer code

Data collection	No particular software was used for data collection
Data analysis	FACS data was analysed with Cellquest Pro 5.2. , Statistical analyses were carried out with Prism version 8

For manuscripts utilizing custom algorithms or software that are central to the research but not yet described in published literature, software must be made available to editors and reviewers. We strongly encourage code deposition in a community repository (e.g. GitHub). See the Nature Research guidelines for submitting code & software for further information.

Data

Policy information about availability of data

All manuscripts must include a data availability statement. This statement should provide the following information, where applicable:
- Accession codes, unique identifiers, or web links for publicly available datasets
- A list of figures that have associated raw data
- A description of any restrictions on data availability

The raw data associated with all of the figures and tables are available on request.

Field-specific reporting

Life sciences study design

All studies must disclose on these points even when the disclosure is negative.

Sample size	The MHRA Target Product Profile (TPP), last updated 15 Oct 2020, specifies testing at least 200 confirmed positive and 200 confirmed negative cases for evaluation of antibody testing devices/platforms. Combining the sets we have described exceeds these requirements (269 samples from PCR+ve donors and 278 negative controls).
Data exclusions	6 of 202 control serum samples from the pre-covid donors in Figure 4 were found to have been given duplicated labels. One pair were negative on HAT, the two other pairs gave disparate but reproducible results, one of the pair positive, one negative. These two pairs giving disparate results were replaced with repeat samples from the original serum stock in the biobank, and retested twice, and gave clear negative results. Thus from the original 202 control samples, we reported on 199 after correcting for these 3 duplicated samples, each of which was scored negative after the repeat testing.
Replication	All the data presented in the paper were found to be reproducible, and the number of replications performed are now indicated in the legend of each figure. Specifically for each figure:<br>1C: Gel was performed twice;<br>2A: 6X, 2B: 3X<br>3A: 2X, 3C: 4X<br>4 and 5: 2X, the repeated results are described in the paper and compared. In both cases the samples were randomised and read independently by two masked assessors. Any disagreements on scoring were resolved by prior agreement to take the lower score of the two.<br>6: performed once in triplicate as a proof of concept<br>Sup data<br>Table S1: Antibodies were titrated at least 3x each<br>S1: 2x<br>S2: repeat of fig. 5<br>Table S2: repeat of Table 1 34 days later<br>Tables S3 and S4: Assays performed once in triplicates on four different blood samples.
Randomization	For Figure 4 the samples were plated after randomisation and delivered without the key. The HAT scores were then read by two independent assessors, and any disagreements resolved by taking the lower score (as agreed prior to the testing). For the repeat, the columns of samples were re-randomised and plated by one individual, and read by a second masked individual. For figure 5 the samples were plated after randomisation and read by masked assessors as above. The serum samples were stored for 34 days in their randomised order, and the whole experiment repeated. Both results are in the paper.
Blinding	For Figures 4 and 5 the samples were randomised and the assessors read the plates without the key which described the PCR results and origin of the samples.<br>Blinding was not done during data collection or analysis of the titration of monoclonal antibodies. These were however reproduced by multiple experimenters.

Reporting for specific materials, systems and methods

We require information from authors about some types of materials, experimental systems and methods used in many studies. Here, indicate whether each material, system or method listed is relevant to your study. If you are not sure if a list item applies to your research, read the appropriate section before selecting a response.

Materials & experimental systems

n/a	Involved in the study	
☐	☒	Antibodies
☐	☒	Eukaryotic cell lines
☒	☐	Palaeontology and archaeology
☒	☐	Animals and other organisms
☐	☒	Human research participants
☒	☐	Clinical data
☒	☐	Dual use research of concern

Methods

n/a	Involved in the study	
☒	☐	ChIP-seq
☐	☒	Flow cytometry
☒	☐	MRI-based neuroimaging

Antibodies

Antibodies used	CR3022, VHH72-Fc, C121, H11-H4-Fc and S309 were produced in house based on published protein sequences.<br><br>Monoclonal antibodies EY-6A, FI-4A, FI-3A, FI-1C, FD-5D, FD-11A, FN-12A, FJ-10B, FM-7B, EZ-7A, EW-8B, EW-9C, and FJ-1C are antibodies isolated in our lab and are described in Huang, et al. (2020). Plasmablast-derived antibody response to acute SARS-CoV-2

	infection in humans. bioRxiv. doi:10.1101/2020.08.28.267526<br>They have been validated in the following studies: ter Meulen et al 2006 Plos Med, Wrapp et al 2020 Cell, Robbiani et al 2020 Nature, Zhou et al 2020 Nat Struct Mol Biol, Pinto et al 2020 Nature and Huang et al 2020 bioxRiv.<br><br>Anti-His tag antibody (Invitrogen, Cat No. MA1-21315; RRID AB_557403)<br><br>Monoclonal antibody to human IgG (Gamma chain specific) Clone GG-5 (Sigma Cat. No. I5885)<br><br>Jackson Alexa Fluor 488-AffiniPure Goat Anti-Human IgA + IgG + IgM (H+L) cat n° JIR109-545-064 .
Validation	Manufacturer's validation statement for anti IgG Clone GG5: Monoclonal anti-Human IgG is immunospecific for human IgG as determined by an ELISA. No reactivity with human light chains of human IgM is observed.<br><br>Manufacturer's validation statement for Jackson Alexa Fluor 488-AffiniPure Goat Anti-Human IgA + IgG + IgM (H+L) : Based on immunoelectrophoresis and/or ELISA, the antibody reacts with human IgA, IgG, and IgM. It also reacts with the light chains of other human immunoglobulins. No antibody was detected against non-immunoglobulin serum proteins. The antibody may cross-react with immunoglobulins from other species.

Eukaryotic cell lines

Policy information about cell lines

Cell line source(s)	Expi293 (RRID CVCL_D615) and ExpiCHO-S (RRID: CVCL_5J31) cell lines were obtained from the Life Technologies.<br>HEK293 cells was obtained from Absolute Antibody Ltd, Oxford)
Authentication	Authentication analysis was not performed for the cell lines used.
Mycoplasma contamination	Original stocks of Expi293F and ExpiCHO cultures were tested to be free of mycoplasma contamination. A new frozen vial was thawed every month to avoid prolonged passaging.
Commonly misidentified lines<br>(See ICLAC register)	None of the 3 cell lines used in the study are listed in the ICLAC register

Human research participants

Policy information about studies involving human research participants

Population characteristics	Samples for Figure 4. Covid Negative serum samples from the Oxford BioBank (OBB) were collected from healthy adults aged 30-50 yrs, 39% male, in the Oxford region of the UK between Sept 4 2014- Oct 4 2016. Anonymised convalescent plasma samples from PCR positive donors were provided by the NHS Blood Transfusion Service from patients at least 28 days post symptom onset. Samples for figure 5: Patients hospitalised with COVID-19 were consented and recruited if they had symptoms and signs consistent with COVID-19 and a positive test result for a nasopharyngeal SARS-CoV-2 reverse transcriptase polymerase chain reaction assay performed in an accredited laboratory between March-April 2020 (median age 64, 59% male). A small number of healthcare worker individuals with mild COVID-19 disease diagnosed using the same tests were included as a comparator group (median age 33, 38% Male). Patients with unselected sepsis (median age 74, 55% male) were identified using the same methods as the hospitalised COVID-19 study but instead identifying patients with signs and symptoms of severe sepsis using the NEWS criteria in the immediate pre-pandemic era (October 2019-March 2020). Healthy volunteers (median age 66, 77% male) were individuals who self-reported as healthy with no recent symptoms or signs of COVID-19 disease.
Recruitment	Figure 4: Pre-covid negative samples from Oxford Biobank were recruited from a regional UK population. Recruitment bias for NHSBT positives - these individuals self-selected to donate blood following convalescence. They therefore represent a group who were likely to have had symptomatic SARS-Cov-2 infection, but recovered sufficiently to nominate themselves for plasma donation. We therefore are less likely to have included cases of trivial or asymptomatic infection (this could artificially inflate our estimates of sensitivity). Again, they reflect a UK population with limited ethnic/geographic diversity. Figure 5: The hospitalised patients were recruited using ethically approved protocols that allowed consenting from nominated consultees and next-of-kin. This was explicitly included to minimise any bias owing to participation attrition owing to disease severity. These patients were recruited in Oxfordshire that represents a predominantly white population and therefore will not represent other major ethnically diverse cities but the controls provided in this study were matched for ethnicity. Healthy volunteers were invited through advertisement to donate blood samples in the early phase of the pandemic. There is no perceived selection bias for healthy volunteers.
Ethics oversight	Oxford Clinical Research Ethics Committee (ref: 08/H0606/107+5); Oxford REC C (ref: 13/SC/0149); Oxford REC C, (ref:19/SC/0296); Cambridge East REC (ref: 20/EE/0101); REMAP-CAP [EudraCT 2015-002340-14]; Sheffield REC (ref: 16/YH/0247] ISARIC/WHO); Scotland A Research Ethics Committee (Ref:534 20/SS/0028).<br>Institutional Human Ethics Committee New Delhi; (Ref.562 IHEC#128/20).

Note that full information on the approval of the study protocol must also be provided in the manuscript.

Flow Cytometry

Plots

Confirm that:

☐ The axis labels state the marker and fluorochrome used (e.g. CD4-FITC).

☐ The axis scales are clearly visible. Include numbers along axes only for bottom left plot of group (a 'group' is an analysis of identical markers).

☐ All plots are contour plots with outliers or pseudocolor plots.

☐ A numerical value for number of cells or percentage (with statistics) is provided.

Methodology

Sample preparation	RBCs were simply washed 3X after HAT, before staining with FITC-labelled polyclonal Anti Human Ig.
Instrument	Beckton Dickinson FACScalibur
Software	Cellquest Pro 5.2
Cell population abundance	As indicated in the manuscript, 5000 Red Blood Cells were analysed for each sample.
Gating strategy	No gating strategy was required. As can be seen on the pdfs provided in sup information we simply used a broad rectangular FSC/SSC gate to eliminate cellular debris or large cell aggregates.

☒ Tick this box to confirm that a figure exemplifying the gating strategy is provided in the Supplementary Information.
